# Knowledge and Attitude Toward Hemoglobinopathy Premarital Screening Program Among Students of Health Colleges at Qassim University

**DOI:** 10.7759/cureus.77081

**Published:** 2025-01-07

**Authors:** Ghaday Almutairi, Ghusun S Al Harbi, Lama Almutairi, Fai S Aljarallah, Nuha M Alzaydi, Rola Alradaddi, Alanoud Alofi, Mariyyah M Alharbi, Norah H Alhumaidi, Tameem A Alhomaid

**Affiliations:** 1 College of Medicine, Qassim University, Unaizah, SAU; 2 Family Medicine, Qassim Health Cluster, Buraidah, SAU

**Keywords:** attitude, hemoglobinopathies, knowledge, premarital screening, qassim university, sickle cell anemia, thalassemia

## Abstract

Introduction:Premarital screening (PMS) for hemoglobinopathies, particularly thalassemia and sickle cell disease, is a public health campaign designed to reduce the prevalence of these genetic illnesses in kids. The purpose of this study was to assess knowledge and attitudes concerning PMS for hemoglobinopathies, including sickle cell anemia and thalassemia, among health college students at Qassim University.

Methods:A cross-sectional methodology was employed with a standardized, self-administered questionnaire examining students' awareness and attitudes toward PMS. Participants' knowledge of sickle cell anemia and thalassemia screening was assessed, as well as their views on marrying someone who has the same genetic illness. Chi-square and analysis of variance (ANOVA) tests were used to assess the relationships between demographic characteristics, knowledge, and attitudes.

Results: This study examined knowledge and attitudes toward hemoglobinopathies PMS among 300 young adults. Participants demonstrated high knowledge levels (mean score 6.93/8, 86.7%) and positive attitudes (mean score 11.46/14, 81.9%). Knowledge was significantly higher among university-educated participants (p < 0.001). While attitudes were generally positive across demographics, older participants (26-44 years) exhibited significantly more positive attitudes than those aged 18-25 (p = 0.008). There is strong support for PMS (94.7%) and public awareness initiatives (>99%) but less agreement on marrying someone with the same genetic trait (62.5%) and less consensus on the significance of seminars (20.8%).

Conclusion: Health college students at Qassim University have a strong understanding of PMS for hemoglobinopathies, with educational level influencing knowledge depth and attitudes. The findings indicate that additional educational programs, notably on the significance of screening in lowering genetic transmission risks, are needed. Raising public awareness through targeted seminars and media initiatives may improve the acceptability and understanding of PMS.

## Introduction

Sickle cell anemia and beta-thalassemia are among the most prevalent hereditary blood disorders globally, posing significant health burdens [1], particularly in regions like the Kingdom of Saudi Arabia, where consanguineous marriages remain common. In Saudi Arabia, consanguineous marriages contribute to the propagation of hemoglobinopathies, leading to ongoing health challenges in the region [2-4]. Both sickle cell anemia, marked by abnormally shaped red blood cells, and thalassemia, which impairs hemoglobin production, result in substantial morbidity and can lead to life-threatening crises if unmanaged [5]. The cultural practice of cousin marriages in Saudi Arabia contributes to the perpetuation of these diseases, as carriers of these genetic disorders may inadvertently pass them on to their offspring [2].

Health college students represent an important demographic for assessing awareness and attitudes toward premarital screening (PMS) for hemoglobinopathies. Studies indicate that while health students may have a foundational knowledge of sickle cell anemia and thalassemia, they often lack a full understanding of how PMS can mitigate the risks associated with these diseases [6,7].

Research demonstrates a positive correlation between higher levels of education and awareness of both the impact of genetic disorders and the value of screening programs [8]. For instance, Omani students with advanced educational levels have been found to express a greater appreciation for seminars and media efforts that promote awareness of genetic disorders, suggesting that populations with limited access to such resources could benefit from targeted educational interventions [9].

In Kuwait, the Saudi Ministry of Health responded by implementing a national PMS program in 2009 aimed at reducing hemoglobinopathy prevalence through carrier screening for couples before marriage [10]. In 2003, the Saudi government had already introduced a mandatory PMS program to reduce the incidence of Saudi Arabia's two most frequent hemoglobinopathies, sickle cell disease and thalassemia [11]. However, a lack of public awareness about the benefits of PMS, particularly in some regions, highlights the need for research to further explore knowledge levels among specific population groups, such as health college students, who are to become future healthcare professionals.

Health college students play a crucial role in disseminating medical knowledge and promoting public health initiatives. This discrepancy may suggest underlying social biases or misconceptions that hinder effective communication of public health policies. Therefore, this study aimed to assess the awareness and attitudes of health college students at Qassim University regarding PMS for hemoglobinopathies. The findings of this research would help address the knowledge gap, offering insights into how well future health professionals understand and support genetic disease prevention. Identifying students' current awareness levels would also help determine which areas may require additional educational efforts to effectively promote PMS and other public health interventions.

## Materials and methods

Study design

This study utilized a cross-sectional research design, which is well-suited for measuring knowledge and attitudes at a single point in time within a specific population. This approach enables efficient data collection and facilitates the identification of patterns and relationships between demographic variables and participants' knowledge levels in the targeted population.

Study population

The study targeted students enrolled in health-related programs (such as medicine, nursing, public health, etc.) at Qassim University, Saudi Arabia. These students were chosen because, as future healthcare providers, they are likely to play a key role in promoting PMS for genetic disorders within their communities.

Sample size

The final sample included 300 participants, chosen through a representative sampling method to ensure that findings could be generalized to similar student populations. This sample size was sufficient to achieve statistical power for testing demographic differences and examining the relationship between demographic factors and knowledge or attitudes toward PMS.

Data collection

Data were gathered using a structured, self-administered questionnaire divided into three sections. The first section gathered data on age, gender, marital status, and education level to assess potential demographic influences on knowledge and attitudes. The second section assessed participants' awareness of hemoglobinopathies, specifically sickle cell anemia and thalassemia, and their knowledge of PMS's role in reducing the transmission of these disorders. Participants were also asked whether they knew that these conditions could be detected through PMS.

The final section examined participants' attitudes toward PMS, including their openness to marrying someone with a genetic disorder and their perception of the importance of public health initiatives (such as seminars and media campaigns) in raising awareness about genetic disorders.

Statistical analysis

Data analysis was conducted using IBM SPSS Statistics for Windows, Version 24 (Released 2016; IBM Corp., Armonk, New York, United States), employing a variety of statistical tests. Descriptive statistics summarized demographic data and responses to knowledge and attitude questions, using frequencies and percentages for categorical data, and means and standard deviations for quantitative data. The Chi-square test was used to explore associations between categorical variables, such as gender and knowledge of specific hemoglobinopathies. Independent sample T-test examined differences in knowledge levels between two groups, such as male and female participants, while one-way analysis of variance (ANOVA) evaluated differences in knowledge and attitude scores across multiple groups, such as those based on educational level. A statistical significance was considered at p < 0.05.

Ethical consideration

This study was approved by the National Committee of Bio & Med. Ethics (NCBE) (Registration No. H-04-Q-001). All participants gave informed consent after being informed about the study's aim, procedures, and their rights, which included the ability to withdraw at any time without penalty. Anonymity and confidentiality were tightly enforced, with all data securely stored and accessible only to the research team to preserve participant privacy.

## Results

Demographic characteristics

Table 1 shows the demographic characteristics of the study participants. The study sample consisted predominantly of young adults, with 263 (87.7%) aged between 18 and 25. The majority of participants were female (236, 78.7%) and had attained a university-level education (249, 83.0%). Most participants reported being single (273, 91.0%), followed by married (24, 8.0%), with a small percentage identifying as divorced (3, 1.0%). 

**Table 1:** Demographic characteristics of the participants

Variables	N	%
Age group		
18-25	263	87.7%
26-35	34	11.3%
36-44	3	1.0%
Gender		
Female	236	78.7%
Male	64	21.3%
Educational level		
Elementary	3	1.0%
High school	48	16.0%
University	249	83.0%
Marital status		
Divorced	3	1.0%
Married	24	8.0%
Single	273	91.0%

Knowledge 

Table 2 shows the descriptive statistics of the knowledge of hemoglobinopathies. The results revealed a high overall level of knowledge regarding hemoglobinopathy PMS. Participants demonstrated a strong understanding of the screening's purpose, recognizing its ability to diagnose both sickle cell anemia and thalassemia. While knowledge was high across all areas, understanding of the screening's role in preventing sickle cell anemia was slightly stronger (mean = 1.79) compared to thalassemia (mean = 1.71). Similarly, participants were slightly more likely to correctly identify the screening's diagnostic capability for sickle cell anemia (mean = 1.76) than for thalassemia (mean = 1.67). Although these differences are minor, they suggest a potential area for focused education, emphasizing the importance of PMS for thalassemia prevention and diagnosis. The overall high average score of 6.93 out of eight (86.7%) indicates a good baseline understanding of the importance of PMS for these genetic conditions.

**Table 2:** Knowledge of hemoglobinopathy premarital screening

Knowledge items	Mean	SD	Knowledge level
Can sickle cell anemia be diagnosed through premarital screening?	1.76	0.520	High
Can thalassemia be diagnosed through premarital screening?	1.67	0.561	High
Does premarital screening reduce the risk of acquiring sickle cell anemia?	1.79	0.499	High
Does premarital screening reduce the risk of acquiring thalassemia?	1.71	0.547	High
Total knowledge score (out of 8)	6.93	1.65	High

**Figure 1:**
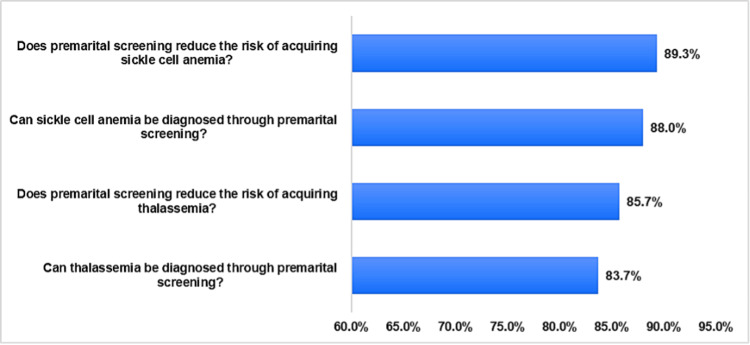
Relative weight of the knowledge of hemoglobinopathies and premarital screening items

In addition, Figure 1 illustrates the relative weight of the knowledge items. High percentages of the participants correctly identified that PMS can reduce the risk of acquiring sickle cell anemia (89.3%) and thalassemia (85.7%). Similarly, a large proportion correctly understood that sickle cell anemia (88.0%) and thalassemia (83.7%) can be diagnosed through PMS.

Comparison of the Knowledge Levels According to the Demographics

Table 3 explores the relationship between demographic factors and knowledge of hemoglobinopathy PMS. Educational level showed a statistically significant association with knowledge scores (p < 0.001). Participants with a university education demonstrated the highest average knowledge score (7.05), followed by those with a high school education (6.54). Participants with only elementary education had a substantially lower average score (3.67). This highlights the importance of educational attainment in understanding PMS for genetic diseases. In addition, age, gender, and marital status were also examined. No statistically significant differences in knowledge scores were observed between these groups. 

**Table 3:** Comparison of the knowledge levels across demographic variables AD: standard deviation; ANOVA: analysis of variance (a) The p-value is calculated by a one-way ANOVA test. (b) The p-value is calculated by an independent t-test. **Significant at < 0.001

Variables	Mean	SD	Test values	p-value
Age group^a^				
18-25	6.98	1.55	f-test = 2.285	0.104
26-35	6.44	2.31		
36-44	8.00	0.000		
Gender^b^				
Female	6.96	1.60	t-test = 0.489	0.625
Male	6.84	1.84		
Educational level^a^				
Elementary	3.67	1.79	f-test = 8.206	<0.001**
High school	6.54	1.69		
University	7.05	1.57		
Marital status^a^				
Divorced	5.67	4.04	f-test= 0.896	0.409
Married	6.92	1.74		
Single	6.95	1.61		

Attitude

Table 4 shows participants' attitudes toward hemoglobinopathy PMS. Overall, attitudes were highly positive, as reflected in the high total attitude score (11.46 out of 14). Participants overwhelmingly expressed willingness to undergo PMS themselves (mean = 1.89) and strongly supported community engagement (mean = 1.99) and the media's role (mean = 1.99) in raising awareness. They also strongly favored the inclusion of medical education in schools (mean = 1.93). However, agreement on the importance of seminars was considerably lower (mean = 0.42). Interestingly, while participants demonstrated a positive attitude toward screening in general, there was only moderate agreement (mean = 1.25) with the statement about marrying someone who also carries the genetic trait. This suggests a more nuanced perspective on personal decision-making related to genetic compatibility and marriage, despite a high overall acceptance of PMS.

**Table 4:** Attitudes toward hemoglobinopathy premarital screening SD: standard deviation

Attitude items	Mean	SD	Attitude level
Will you undergo premarital screening?	1.89	0.411	High
If you have the genetic trait, would you marry someone who also carries this trait?	1.25	0.776	Moderate
Do you agree on the importance of seminars?	0.42	0.691	Low
Do you agree on the importance of medical education in schools?	1.93	0.256	High
Would you encourage the community to participate in raising awareness?	1.99	0.115	High
Do you believe in the importance of media's role in raising awareness?	1.99	0.082	High
Do you recognize the significance of media in enhancing public awareness?	1.99	0.100	High
Total attitude score (out of 14)	11.46	0.97	High

**Figure 2:**
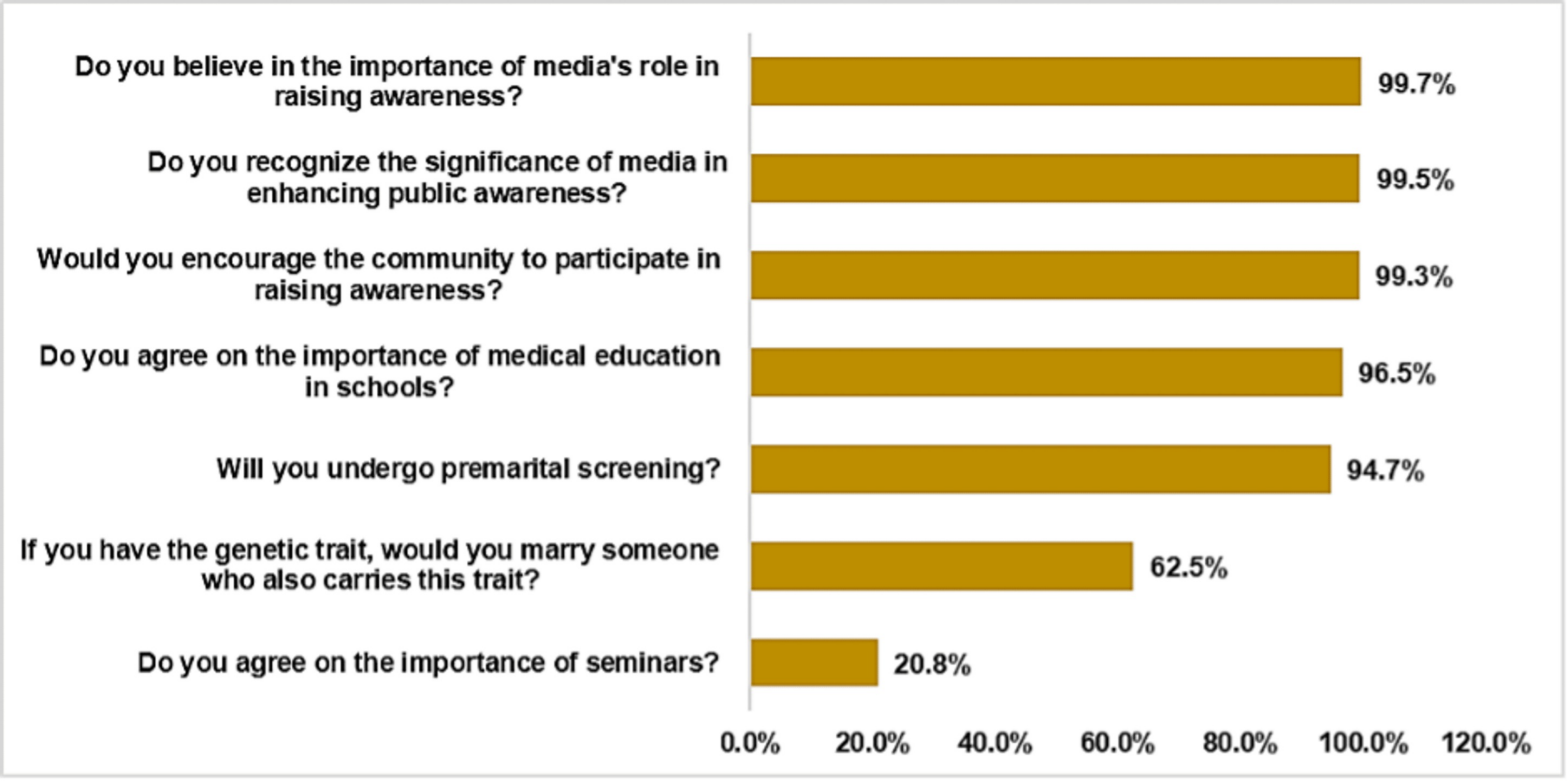
Relative weight of the attitudes toward hemoglobinopathy premarital screening and awareness initiatives

In addition, Figure 2 illustrates the relative weight of the attitude items. The vast majority of participants expressed a willingness to undergo PMS (94.7%) and strongly supported community participation (99.3%) and the media's role (99.7% and 99.5%) in raising awareness about hemoglobinopathies. A high percentage also agreed on the importance of medical education in schools (96.5%). However, 62.5% indicated they would marry someone carrying the same genetic trait, and a markedly lower percentage (20.8%) agreed on the importance of seminars as an awareness strategy.

Comparison of the Attitude Levels According to the Demographics

Table 5 provides an analysis of attitude levels across different demographic variables. The age group showed a statistically significant difference (p = 0.008) in attitude levels, with older age groups exhibiting higher mean scores. However, no significant differences were observed in attitude levels based on gender, educational level, or marital status.

**Table 5:** Comparison of attitude levels across demographic variables SD: standard deviation; ANOVA: analysis of variance (a) The p-value is calculated by a one-way ANOVA test. (b) The p-value is calculated by an independent t-test. **Significant at < 0.01

Variables	Mean	SD	Test values	p-value
Age group^a^				
18-25	11.40	0.931	f-test = 4.97	0.008**
26-35	11.79	1.09		
36-44	12.67	1.15		
Gender^b^				
Female	11.49	0.925	t-test = 0.940	0.348
Male	11.36	1.10		
Educational level^a^				
Elementary	10.67	3.51	f-test = 2.50	0.083
High school	11.25	1.06		
University	11.51	0.890		
Marital status^a^				
Divorced	10.33	2.89	f-test = 2.15	0.118
Married	11.54	1.18		
Single	11.47	0.911		

## Discussion

PMS for hemoglobinopathies, particularly thalassemia and sickle cell disease, is a public health initiative aimed at reducing the incidence of these genetic disorders in offspring. This screening is particularly relevant in regions where these conditions are prevalent, such as parts of the Middle East, including Saudi Arabia [8,11,12]. PMS is a public health measure that identifies carriers of hemoglobinopathies before marriage, reducing the prevalence of these genetic disorders through informed reproductive choices [12,13]. However, its acceptance varies across cultural and religious contexts, with legal and religious factors affecting its implementation. Educational initiatives are crucial to overcome misconceptions and promote the benefits of screening, ensuring that couples are aware of potential risks to their future children [14]. Our study evaluated knowledge and attitude toward hemoglobinopathy PMS programs among students of health colleges at Qassim University, as these are expected to drive future interventions against hemoglobinopathies in Saudi Arabia. 

The findings show that health college students have a reasonable understanding of PMS tests for hemoglobinopathies, specifically sickle cell anemia and thalassemia. Overall, a high average score of 6.93 out of eight (86.7%) indicates good baseline knowledge. This is better knowledge than the knowledge reported among the general population in Riyadh, showing that the overall knowledge level of the participants was 35.8% good, 35.1% moderate, and 29.1% bad knowledge [8]. Moreover, studies conducted in Turkey, one of the countries with a high prevalence of sickle cell anemia and thalassemia, revealed that 57.7% of participants had prior knowledge about thalassemia and its screening programs, with 91% acknowledging PMS's importance in reducing genetic disease prevalence [12,14]. Primary sources of information were media outlets, but 53.2% mistakenly considered the trait an outright disease rather than a carrier status [12]. Our findings show better knowledge than the general couples in Riyadh, Saudi Arabia, and Turkey, which reflects highly on the education these students have received, most likely as a result of the health-related curriculum they have completed. Another Qatari study found that knowledge of PMS was significantly correlated with students enrolled in a health-related college and a participant's parents' non-consanguineous marriage [6]. This is also supported by the findings from Muscat, Oman, where most high school students (78.1%) were also aware of the availability of the PMS program, 87.4% believed that PMS is important, and most students (87.2%) indicated that they would undergo the screening [9], and their main source of information was family and friends (34.3%) [9]. Though our participants had good knowledge, there are significant gaps, particularly in terms of how PMS can reduce the likelihood of transferring genes associated with these illnesses. Although most students are aware that screening is available, their awareness of its specific function in risk reduction appears to be limited, emphasizing the need for further education.

We found no significant gender differences in knowledge about PMS for sickle cell anemia and attitudes, showing that both male and female students understand the public health implications. This equality in knowledge between genders inside health colleges is encouraging, implying that gender differences in health information, which have been emphasized in other studies [15,16], may be less significant in this setting. Contrasting our findings, a Qatari study found that the female gender was significantly associated with better knowledge of PMS [6], and this might explain why a study in Oman found that females were significantly more supportive of making PMS mandatory (p = 0.002) and enforcing PMS regulations (p = 0.010) than males [9]. Though we could not find significant differences in knowledge and attitude based on education, other previous studies conducted in Saudi Arabia, Turkey, and Qatar also found a significant relationship between awareness and educational level, indicating that college-educated participants had the highest level of knowledge [5,6,8]. This may explain why 96.5% of our participants agreed on the importance of medical education in schools.

Unlike gender, educational level significantly influences knowledge and attitudes. One-way ANOVA tests on awareness of PMS and the decrease of sickle cell anemia risk show that higher educational levels were associated with a better comprehension of screening's preventative potential. However, variations were found in specific attitudes, such as beliefs about marrying people with similar genes and the necessity of attending seminars. These findings highlight the importance of focused awareness initiatives, particularly in high schools, to ensure that students from all demographics understand the risks and advantages of PMS. 

Consistent with previous studies [6,9], the majority of participants reported a desire to undergo PMS, highlighting genetic screening as an effective health promotion method for controlling hemoglobinopathies in high-prevalence locations. This aligns with another study conducted in Riyadh, showing that 96.1% support and accept the screening program in Saudi Arabia, along with 92.38% of participants who had a positive attitude toward the program [8]. However, 62.5% in our study said they would marry someone who had the same genetic illness, which could imply less discrimination against hemoglobinopathy trait carriers. This agrees with another study conducted in Saudi Arabia, showing that among the 2,375 high-risk couples screened, 89.6% married each other despite the known high-risk status [11]. A previous study in Riyadh also showed that 37.4% of participants considered canceling marriages if given incompatible screening results [6], which is almost a half percentage of our sample willing to cancel marriage. While our findings show a good attitude toward undergoing the screening, other studies showed that students and the general population go a step further. In Oman, around half of the university students (n = 313; 53%) supported making PMS a mandatory practice before marriage, and around one-third (n = 212; 36%) supported enacting rules and regulations to prevent marriage in the event of positive results [17], while females are in favor of enforcing PMS laws (p = 0.010) compared to males [9].

Most students recognized the effectiveness of media and favored the inclusion of medical education in schools to promote awareness about genetic illnesses. This finding implies that students value media campaigns and medical education as helpful means of raising knowledge about PMS and other genetic illnesses. Previous research conducted in Saudi Arabia and neighboring countries also supported the idea of scaling up educational campaigns to raise awareness [6,18,19], supporting our findings. National awareness campaigns must be implemented to enhance knowledge and attitudes among students and the general public concerning PMS [18]. A previous study conducted among female students at King Abdulaziz University (KAU) found that their knowledge about PMS was generally low before the educational campaign. However, after the program, students' knowledge significantly improved, with a mean score of 18.45 ± 4.96 in the post-test (p < 0.001), indicating the program's success, which underlines the need for similar educational programs and incorporating the screening program into secondary and university education curriculums [19].

The practice of cousin marriage, particularly in regions like the Middle East, is deeply rooted in cultural, social, and familial traditions [20]. While it is perceived as a way to strengthen family bonds and preserve wealth within lineages, it also carries significant medical implications, particularly regarding the transmission of genetic disorders such as thalassemia and sickle cell disease [20,21]. Given the intertwined nature of cultural heritage and public health, education campaigns aimed at promoting PMS for hemoglobinopathies must be designed with cultural sensitivity and respect for tradition. Education campaigns should involve community leaders, religious figures, and elders who hold influence over cultural practices. These stakeholders can help frame public health messages that align with cultural values, reducing resistance and fostering acceptance. 

Narrative-based education through storytelling can be a powerful tool involving sharing stories of families affected by genetic disorders due to consanguineous marriages while highlighting successful outcomes from PMS [22]. Campaigns can highlight the positive aspects of cultural traditions while introducing modern medical practices as a means of safeguarding future generations. For example, framing PMS as a way to honor family health and continuity can resonate with cultural values. This has to be coupled with the use of language that respects cultural norms and avoids stigmatizing cousin marriage. Integrating PMS education into school and university curricula can help normalize the practice among younger generations. Topics could include the science of genetic inheritance and the role of PMS in preventing hereditary diseases [23,24]. Media campaigns should feature culturally relevant figures, such as respected community members or celebrities, to endorse PMS, using short, engaging videos or infographics explaining the science behind genetic disorders and the benefits of screening. Training young adults who have undergone PMS to serve as peer educators can create a ripple effect within communities. Moreover, in countries like Saudi Arabia, where religion plays a central role in public life [25], collaboration with religious authorities can enhance the legitimacy of PMS.

When examining the demographic factors associated with attitudes, the age group showed statistically significant differences in attitude levels, with older age groups exhibiting higher mean scores. This suggests that attitudes may be influenced by age-related factors or experiences and aligns with a previous study conducted in Jazan, Saudi Arabia [4]. However, no significant differences were observed in attitude levels based on gender, education level, or marital status, indicating that these factors do not play a substantial role in shaping attitudes within this sample. 

This study has some limitations that could affect its generalizability and depth of findings. The study's cross-sectional design limits the ability to infer causality between variables such as education level and knowledge or attitudes. Longitudinal studies could provide deeper insights into how knowledge and attitudes evolve over time. The sample size was limited to 300 participants, which may not reflect the knowledge and attitudes of students in non-health-related fields or the general population. The self-administered questionnaire may have led to inaccuracies due to misinterpretation or lack of attention from respondents. The study also did not assess the impact of interventions, such as educational campaigns or media initiatives, on knowledge and attitudes. Participants may have provided responses they believed were socially acceptable, especially regarding their attitudes toward PMS rather than their genuine beliefs, leading to social desirability bias. The study primarily focused on demographic factors such as age, gender, education level, and marital status. Other influential factors, such as cultural or familial beliefs and personal experiences with genetic disorders, were not explored. The sample was predominated by female and young participants, which could have affected the results, and this limits the generalizability to male Saudi students and those in middle and advanced age.

To mitigate these limitations, future studies could include participants from diverse gender, educational, and cultural backgrounds, use longitudinal designs to assess changes in knowledge and attitudes over time, use mixed methods to combine quantitative surveys with qualitative interviews, expand the scope of variables to include cultural, familial, and societal influences, and evaluate the effectiveness of targeted interventions on knowledge and attitudes. Moreover, the use of a specific questionnaire would have been more effective with more specific questions targeted depending on the sample types.

## Conclusions

This study established that health college students at Qassim University possess a strong level of knowledge regarding PMS for hemoglobinopathies, including sickle cell anemia and thalassemia. However, gaps remain, particularly in understanding how screening can reduce genetic vulnerability. There is little variation in knowledge between genders, indicating that both male and female students are equally informed. Educational level, however, does influence both knowledge and attitudes: higher education correlates with greater knowledge and a more cautious attitude toward genetic risk factors in marriage. Additionally, the findings highlight students' positive attitudes toward PMS and their recognition of seminars and media as valuable tools for raising awareness. These results underscore the need to continue educational initiatives and awareness campaigns for PMS in the region and further studies to keep informing interventions. Education campaigns related to PMS and cousin marriage must navigate the delicate balance between public health imperatives and cultural traditions. By adopting culturally sensitive approaches, leveraging community engagement, and addressing knowledge gaps, these campaigns can empower individuals to make informed decisions while respecting their heritage. The success of such initiatives will depend on collaboration between healthcare professionals, educators, religious leaders, and policymakers, ensuring that the benefits of PMS are accessible and acceptable to all.
